# Multi-surface analysis for human action recognition in video

**DOI:** 10.1186/s40064-016-2876-z

**Published:** 2016-08-02

**Authors:** Hong-Bo Zhang, Qing Lei, Bi-Neng Zhong, Ji-Xiang Du, Jialin Peng, Tsung-Chih Hsiao, Duan-Sheng Chen

**Affiliations:** Department of Computer Science and Technology, Huaqiao University, Fujian, China

**Keywords:** Human action recognition, Multi-view video analysis, Three surfaces motion feature, Probability inference

## Abstract

The majority of methods for recognizing human actions are based on single-view video or multi-camera data. In this paper, we propose a novel multi-surface video analysis strategy. The video can be expressed as three-surface motion feature (3SMF) and spatio-temporal interest feature. 3SMF is extracted from the motion history image in three different video surfaces: horizontal–vertical, horizontal- and vertical-time surface. In contrast to several previous studies, the prior probability is estimated by 3SMF rather than using a uniform distribution. Finally, we model the relationship score between each video and action as a probability inference to bridge the feature descriptors and action categories. We demonstrate our methods by comparing them to several state-of-the-arts action recognition benchmarks.

## Background

Human action recognition in video sequences is a challenging research topic in computer vision (Aggarwal and Ryoo 2011; Poppe 2010) and serves as a fundamental component of several existing applications such as video surveillance human computer interaction, multimedia event detection and video retrieval. Extensive efforts have been devoted to action recognition, including: finding a robust, stable, discrimination feature to represent the action/video, such as the motion history image (MHI), motion trajectories of human bodies (Yoon et al. 2014), and spatio-temporal interest point (STIP) (Dawn and Shaikh 2015), and using effective machine learning or pattern recognition methods to identify human action, such as latent support vector machine (SVM) (Zhou et al. 2015), deep learning (Charalampous and Gasteratos 2014) and statistical methods. Facing complex scenes, action recognition in the depth video and multi-camera systems have gained increasing attention in recent years. However, in practical applications and real scenes, such models are not sufficient due to the variations in multiple facets and their high computational cost.

Modeling human actions in hybrid data, such as recognizing an action in RGB-depth data, multi-camera view data and mixed data, is one effective method for human action recognition in complex and dynamic environments. Many works have demonstrated the superior performance obtained when using hybrid data compared to a single data source. For example, Luo et al. (2013) proposed a framework for the real-time realization of human action recognition in distributed camera networks. Liu et al. (2015) proposed the pyramid partwise bag of words (PPBoW) representation and regarded single/multi-view human action recognition as a multi-task learning problem penalized by the graph structure. Due to the limitations of devices, these methods have many restrictions in real scenes and high computational complexity.

**Fig. 1:**
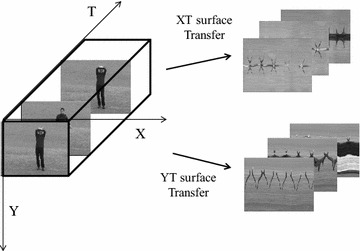
Sketch of the multi-surface transfer of video. *X* indicates the *horizontal* direction, *Y* indicates the *verticality* direction and *T* indicates the time direction. The image sequence in the *top right* corner is the result of *XT* surface transfer. And the image sequence in the *lower right* corner is the result of *YT* surface transfer

In contrast to previous studies, we find that motion can be represented from multi-surfaces in a signal-view video. The video is expressed based on three surfaces: horizontal–vertical (XY surface), horizontal-time (XT surface) and vertical-time surface (YT surface), as shown in Fig. 1. From the different surfaces, the motion history is extracted and represented as a histogram of the orient gradient (HOG) features to model the holistic action, composing the three-surface motion feature (3SMF). Meanwhile, the STIP feature is extracted to represent the local motion.

**Fig. 2:**
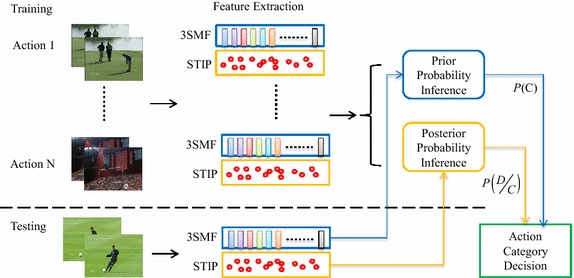
Outline of the workflow of the proposed approach. The 3SMF and STIP feature have been extracted in the training data and testing data. In training process, prior probability inference model is trained by SVM, and posterior probability is estimated by NBNN algorithm

However, the fusion of direct features is not sufficient or robust. To this end, we propose to integrate the holistic features and STIP features into an action classifier. A probability inference model is used to identify the action in the video. The proposed multi-surface video analysis method is shown in Fig. 2. In the training stage, a SVM classifier is trained by 3SMF to estimate the prior probability. The STIP feature is extracted, and a naïve Bayes nearest neighbor algorithm (NBNN) is used to estimate the posterior probability. In the testing stage, the test video is also represented as 3SMF and STIP. The action category is determined by probability inference using the prior probability and posterior probability.

The contributions of our work are threefold:

1. We propose a novel multi-surface video analysis strategy that is different from using multiple cameras.
2. We propose a probability method to combine the holistic and local features. In contrast to the majority of previous works, we use 3SMF for the prior probability rather than the uniform distribution.
3. The experimental results show that the proposed method is effective, robust to scene motion and provides accurate results.

The remainder of this paper is organized as follows. “Related work” section introduces the related works of human action recognition. “Algorithm in the proposed method” section describes the algorithms on which the proposed method is based. “Experimental results and analysis” section presents and discusses the experimental results. “Conclusion” section concludes the work.

## Related work

The numerous existing methods for recognizing human action from image sequences or video have been classified as template-based approaches, local-feature-based approaches (including appearance and motion features) and object/scene-context-based approaches. Methods of human action recognition in multi-view scenes have been proposed in many studies. A literature review (Aggarwal and Ryoo 2011; Poppe 2010; Dawn and Shaikh 2015; Nissi Paul and Jayanta 2016; Paul and Singh 2014) indicated that the work related to our method includes human action recognition approaches based on a STIP detector, human action recognition approaches with multi-view cameras and human action approaches using object/scene context information.

The STIP detector captures the 3D Harris interest points from a video in the spatio-temporal domain, which was extended from the Harris corner detection by Laptev (2005a). The STIP detector is widely used in human action recognition tasks due to its robustness and good performance. Chakraborty et al. (2012) proposed a novel action recognition algorithm using selective STIPs. Yu et al. (2012) developed a spatial–temporal implicit shape model (STISM) for characterizing the space–time structure of sparse local features. Yan and Luo (2012) proposed a new action descriptor, named the histogram of interest point locations, based on STIPs. Yuan et al. (2011a) proposed the naïve Bayes mutual information maximization (NBMIM) algorithm based on STIPs for classifying actions. Zhang et al. (2013) proposed an improved version using the *ε*-NN probability estimation method and the variance filter for discriminative STIP selection. In the proposed method, the *ε*-NN probability estimation method is used in the NBNN algorithm for the posterior probability.

Multi-camera systems can provide more information for action recognition. Liu et al. (2015) proposed a unified single/multi-view human action recognition method via regularized multi-task learning. Gao et al. (2015) proposed a multi-view discriminative and structured dictionary learning method with group sparsity and a graph model to fuse different views and recognize human actions. Junejo et al. (2011) presented an action descriptor to capture the structure of the temporal similarities and dissimilarities in action sequences. The latent kernelized structural SVM was proposed by Wu and Jia (2012) for view-invariant action recognition. These methods of multi-views have good performance. However, the methods cannot be applied to real scenes due to the high computation complexity and difficultly in correlating information among different views.

The methods discussed above are independent of human action recognition. The concept of using context information for action recognition has been widely adopted in recent studies. Object detection and pose estimation play important roles in the process of recognizing human action. Yao and Fei-Fei (2012) proposed a mutual context model to jointly model objects and human poses in human–object interaction activities. Ikizler-Cinbis and Sclaroff (2010) proposed an approach for human action recognition that integrates multiple feature channels from several entities, such as objects, scenes, and humans. Burghouts et al. (2014) used object tracking trajectories as the context for improving threat recognition. Marszalek et al. (2009) proposed the context of natural dynamic scenes for action recognition. The scene information of the video was extracted from the movie script rather than from image sequences. Similarly, in the proposed method, 3SMF is regarded as the context information of the STIP to recognize human action. In the proposed method, 3SMF and STIP are extracted to model action. A probability inference algorithm is used to determine the action categories.

## Algorithms in the proposed method

### STIP and 3SMF features

In recent studies, many local features have been successfully used for human action recognition, such as STIP, dense sample, and dense trajectories (DTs). Numerous studies have demonstrated the good performance and robustness of STIP features. In the proposed method, the STIP is extracted by the 3D-Harris detector proposed by Laptev and Lindeberg (2006), and is described by concatenating the HOG and HOF features (162-dimensional feature vector).

To calculate STIP, the video is constructed a spatio-temporal scale-space representation *L* by convolution with spatio-temporal Gaussian kernel. The second-moment matrix *μ* of spatio-temporal scale-space representation *L* is calculated, which is 3-by-3 matrix composed by first order spatial and temporal derivatives. The response function H is defined by combining the determinant and the trace of *μ* as following:

1 $$\begin{aligned} H & = \det (\mu ) - k \times trace^{3} (\mu ) \\ & = \lambda_{1} \times \lambda_{2} \times \lambda_{3} - k \times (\lambda_{1} + \lambda_{2} + \lambda_{3} ) \\ \end{aligned}$$

where *λ*_1_, *λ*_2_, *λ*_3_ is the eigenvalue of matrix *μ*. The STIP is defined by searching the maxima of the point with *H*. In Laptev’s work, the parameter *k* is set to .005 through experimental results.

**Fig. 3:**
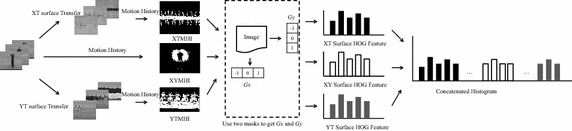
Framework of the 3SMF. Firstly, video is regarded as three different surface image sequences. The MHI is calculated by frame difference. And HOG feature is detection for MHI

To describe the action, we propose a new action feature named the 3SMF. The framework of 3SMF is shown in Fig. 3. The 3SMF is a fusion of the features of three different surfaces and is represented by the HOG feature of the MHI. In the proposed method, STIP feature is regarded as local feature, and 3SMF is holistic feature to represent action. And a probability inference model is used to combine STIP feature with 3SMF feature.

For the input video, the original image sequence appears as an XY surface image sequence $V_{xy} = f(x,y,t)$, where $x \in \{ 1, \ldots ,N_{x} \} ,\,y \in \{ 1, \ldots ,N_{y} \} ,\,t \in \{ 1, \ldots ,N_{x} \} .$*N*_*x*_ is the width of the video, *N*_*y*_ is the height of the video, and *N*_*t*_ is the length of the video. The XT surface image sequence $V_{xt} = f(x^{\prime } ,y^{\prime } ,t^{\prime } )$ is regarded as the original image sequence rotated 90° along the X direction. Similarly, the YT surface image sequence $V_{yt} = f(x^{\prime \prime } ,y^{\prime \prime } ,t^{\prime \prime } )$ is regarded as the original image sequence rotated 90° along the Y direction. The XT and YT surface transfer are expressed by Eq. (2)

2 $$\begin{aligned} & \left( {x^{\prime } ,y^{\prime } ,t^{\prime } } \right) = (x,y,t)\left[ {\begin{array}{ccc} 1 &\quad 0 &\quad 0 \\ 0 &\quad {\cos \alpha } &\quad {\sin \alpha } \\ 0 &\quad { - \sin \alpha } &\quad {\cos \alpha } \\ \end{array} } \right]\quad \alpha = \beta = 90^\circ \\ & \left( {x^{\prime\prime} ,y^{\prime\prime } ,t^{\prime\prime } } \right) = (x,y,t)\left[ {\begin{array}{ccc} {\cos \beta } &\quad 0 &\quad { - \sin \beta } \\ 0 &\quad 1 &\quad 0 \\ {\sin \beta } &\quad 0 &\quad {\cos \beta } \\ \end{array} } \right] \end{aligned}$$

For the XY, YT and XY surface image sequences, the MHI (Ahad et al. 2012) is extracted for the action description. The MHI approach is a view-based temporal template method that is simple yet robust in representing movements and has been widely employed by many researchers for human action recognition, motion analysis and other related applications. Video is expressed as XYMHI, the XT surface image sequence is expressed as XTMHI and the YT surface image sequence is expressed as YTMHI.

**Fig. 4:**
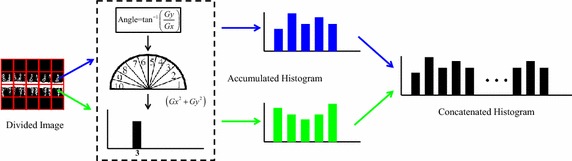
Framework of the HOG feature. The MHI is divided to *M* × *N* grids. The gradient of pixel casts a weight vote for an orientation-based histogram

The HOG feature is computed to represent the MHI. HOG was first developed for use in human detection; the method divides an image into small spatial regions called cells. A local histogram of the gradient direction over the pixels in the cell is constructed. Figure 4 shows the framework of the HOG feature. To calculating HOG feature, it contained four steps: divided image into block by rectangle partitioning, calculated image gradient using two masks ([−1, 0, 1] and [1, 0, −1]), accumulated histogram for each blocks, and concatenated block histogram.

Rectangle partitioning is the most common method for representing small spatial regions in an image. An image can be divided into several rectangles of the same size. The ratio of the block size to the image size typically depends on the total number of blocks. In other words, if an image is divided into *M* × *N* blocks, the block size is $h/M \times w/N,$ where *h* and *w* are the image height and image width, respectively. Traditional block-partition divides an entire image into a grid, in which all blocks are the same size. The block size is crucial because a large block may enclose a contiguous region and produce conspicuous features, whereas a small block cannot adequately represent object characteristics. In this work, both *M* and *N* are set to 9.

The most common gradient computation method is to apply a mask in both the horizontal and vertical directions. This study uses two masks to filter the intensity data of an image to obtain the orientation (or angle) of the current pixel.

Each pixel within a block then casts a weighted vote for an orientation-based histogram channel based on the values calculated by gradient computation. The histogram channels are evenly spread over 0°–180° or 0°–360°. In this work, angles of 0°–180° are divided into ten 18° intervals. To increase the tolerance for vertical and horizontal angles, angles of 0°–9° and 171°–180° are set to the same interval; the angles of 81°–99° form a new interval. After partitioning, feature extraction is applied to construct a local feature histogram for each block, which is concatenated to form the image representation. For a consistent measure, each value for bin *i*, *h*(*i*), is normalized to $h^{\prime } (i)$ within the range of 0–1 by the following equation:

3 $$h^{\prime } (i) = \frac{h(i)}{{\sum\nolimits_{i = 1}^{n} {h(i)} }}$$

where *n* is the total number of bins, i.e., ten in this work. So, the HOG feature length of MHI is 810.

Finally, the HOG feature of each block is concatenated to build the 3SMF feature, and the length of the 3SMF feature is 2430.

The 3SMF feature detection algorithm is summarized in Algorithm 1.

**Table:** Algorithm 1 Three-surface motion feature (3SMF) detection algorithm

Input: Video or Image sequence *V*_*xy*_
Output: Feature Vector *F*
1. Image sequence transfer using Eq. (2): $V_{xy} \to V_{xt} ,$ $V_{xy} \to V_{yt}$
2. For each image sequence, calculate the motion history image (MHI) using frame difference method: $V_{xy} \to XYMHI,$ $V_{xt} \to XTMHI,$ $V_{yt} \to YTMHI$
3. For each MHI image *I*:
(a) Divided into *M* × *N* blocks
(b) Calculated the gradient of all pixel in *I*
(c) Each pixel within a block casts a weighted vote for an orientation-based histogram: $h(i),\,i = 1 \ldots M \times N$
(d) Concatenated the histogram of blocks to represent MHI: $H_{I} = \{ h(1), \ldots ,h(i), \ldots ,h(M \times N)\}$
4. Concatenated MHI feature to build 3SMF feature: $F = \{ H_{xy} ,H_{xt} ,H_{yt} \}$

### Action inference algorithm

To classify the test video *V*, the class of *V* is the class c* that has the maximum probability score between *V* and a specific class *c* corresponding to the following equation:

4 $$c^{*} = \mathop {\arg \hbox{max} }\limits_{{c \in \{ 1, \ldots,\,N_{c} \} }} p(c,V) = \mathop {\arg \hbox{max} }\limits_{{c \in \{ 1, \ldots,\,N_{c} \} }} p(c)p(V|c)$$

where *N*_*c*_ is the number of action categories. Given the prior *p*(*c*) and posterior $p(V|c),$ we can infer the best c* by maximizing the joint distribution *p*(*c*, *V*). Here, we train the SVM classifier to inference the prior *p*(*c*) using the 3SMF feature. The posterior $p(V|c)$ is solved using the NBNN algorithm.

The SVM classifier is a binary classifier in a high-dimensional hyper plane and it is a decision function in high-dimensional space. For the problem of multiclass classification, one-versus-one strategy is used in SVM model training. We build the binary classifier with RBF kernel for every two actions [total of $\frac{{N_{c} \times (N_{c} - 1)}}{2}$ SVM classifiers]. For testing data, the target is to choose the class that is selected by most classifiers. In the training process, fivefold cross-validation is used to find the best parameters of RBF kernel.

To compute the posterior $p(V|c),$ the video is expressed as the set of STIPs $V = \{ d_{v} |v = 1, \ldots ,N_{v} \} ,$ where *d*_*v*_ is the STIP feature and *N*_*v*_ is the number of STIPs. The probability $p(V|c)$ is transformed to the probability $p(d_{1} , \ldots ,d_{{N_{v} }} |c)$ between the STIP and action category. In Native Bayes algorithm, the joint probability $p(d_{1} , \ldots ,d_{{N_{v} }} |c)$ is transformed to the product of each STIP based on independence assumption as following:

5 $$p\left( {d_{1} , \ldots ,d_{{N_{v} }} |c} \right) = \prod\limits_{{N_{v} }} {p\left( {d_{v} |c} \right)}$$

And to calculate the probability $p(d_{v} |c),$ Gauss probability distribution is used based on nearest neighbors.

For a special video, NBNN approximation is used to estimate the probability as follows:

6 $$\begin{aligned} p\left( {V|c} \right) & = \prod\limits_{{N_{v} }} {p\left( {d_{v} |c} \right)} \\ p\left( {d_{v} |c} \right) & = \frac{1}{{\left| {NN_{\varepsilon }^{{c_{i} }} \left( {d_{v} } \right)} \right|}}\sum\limits_{{d_{t} \in T^{{c_{i} }} }} {K\left( {d_{v} - d_{t} } \right)} \\ & \approx \frac{1}{{\left| {NN_{\varepsilon }^{{c_{i} }} \left( {d_{v} } \right)} \right|}}\exp \left[ { - \frac{1}{{2\sigma^{2} }}\left( {\left\| {d_{v} - d_{NN} \left( {d_{v} } \right)} \right\|^{2} } \right)} \right] \\ \left\| {d_{v} - d_{NN} \left( {d_{v} } \right)} \right\| & = \mathop {\hbox{min} }\limits_{{d_{t} \in NN_{\varepsilon }^{{c_{i} }} \left( {d_{v} } \right)}} \left\| {d_{v} - d_{t} } \right\|, \\ \end{aligned}$$

where $T^{{c_{i} }}$ is the STIP set of the training data with action label *c*_*i*_. $NN_{\varepsilon }^{{c_{i} }} (d_{v} )$ denotes the set of samples *d*_*t*_ in the training videos $T^{{c_{i} }}$, with distances to *d*_*v*_ of less than *ε*. Furthermore, $d_{NN} (d_{v} )$ is the set of nearest neighbors of *d*_*v*_ in the set $NN_{\varepsilon }^{{c_{i} }} (d_{v} ).$ Recognition performance is insensitive to the choice of *ε*. The experimental results of Yuan et al. (2011b) and Zhang et al. (2013) have shown that setting *ε* to 2.2 yields the highest accuracy. The same conclusion was obtained in this work; thus, we set *ε* to 2.2.

The proposed action recognition approach is summarized in Algorithm 2.

**Table:** Algorithm 2: Action recognition through multi-surface analysis

Input: Video or Image sequence *V*_*xy*_
Output: Action category c*
Training:
1. Detection STIPs for training data: $T = \cup T^{{c_{i} }}$
2. Detection 3SMF using Algorithm 1 for training data
3. Using 3SMF feature to train SVM model
Testing:
1. Detection STIPs for testing data $D = \{ d_{v} |v = 1, \ldots ,N_{v} \}$
2. Detection 3SMF feature for testing data
3. For each feature *d*_*v*_ in *D*, searching nearest neighbors in the STIP set of training data, and calculate the probability $p(V|c)$
4. Using SVM model to calculate the prior probability *p*(*c*)
5. Inference action by Eq. (4)

### Computational complexity

In the initial of the test process, the 3SMF and STIP features are extracted from the input video. The computation of these features need iterate over all of pixels in the video, so the computation complexity of the feature extraction is $N_{x} \times N_{y} \times N_{t} .$ The SVM algorithm can calculate the classification probability of test feature in linear time. The intensive computation for NBNN is to search nearest neighbors from training data for all of the STIP features extracted from test video. The computation complexity of searching nearest neighbors depend on the size of training set. In the experiments, the number of STIP features extracted from training set is more than hundreds of millions. The computational complexity of the proposed is combination of feature detection and nearest neighbors searching in training data. However, due to the STIP set of training data is large, and the method of 3SMF detection, unfortunately, the proposed method is not suitable for real-time recognition.

## Experimental results and analysis

### Action dataset

**Fig. 5:**
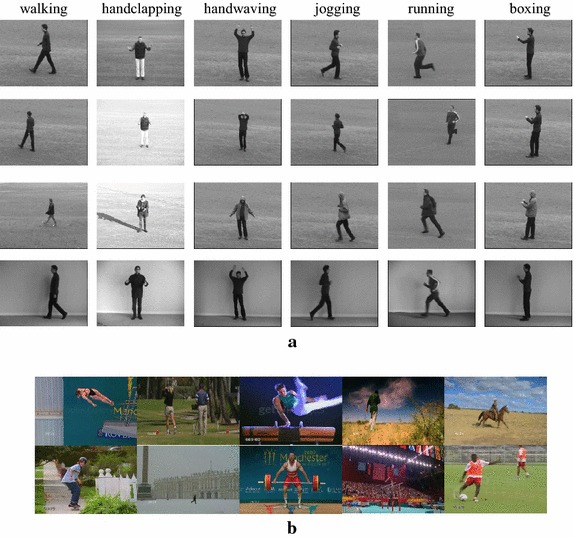
Examples of action datasets. **a** KTH dataset, **b** UCF sport dataset

This section describes the experiments used to verify the effectiveness of the proposed methods, as described in “Algorithms in the proposed method” section. All experimental results are obtained using the KTH dataset and UCF sport dataset (Rodriguez et al. 2008). Figure 5 shows some examples from the dataset.

The KTH dataset contains six (K = 6) actions (i.e., walking, jogging, running, boxing, hand-waving, and handclapping). Each action has 25 subjects in four environments (i.e., outdoors, outdoors with variable scales, outdoors with different clothes, and indoors with lighting conditions). Subjects are selected randomly, and their corresponding actions are collected as a training dataset; the remaining videos are used as the dataset test. In our experiment, we used 25-fold leave-one-out cross-validation to measure the performance of the proposed method.

The UCF Sports Action dataset consists of ten different types of sports actions (A = 10), i.e., ‘swing-bench’, ‘swing-side’, ‘diving’, ‘kicking’, ‘lifting’, ‘riding horse’, ‘running’, ‘skateboarding’, ‘golf swing’, and ‘walking’. The dataset consists of 150 real videos. A horizontally flipped version of each video sequence was added to the dataset to increase the number of training samples. In our experiment, we used the leave-one-out strategy to test each original action sequence, whereas the remaining original video sequences, together with their flipped versions, were included in the training set.

In the experiment, certain parameters affect the accuracy of action recognition. The relevant parameters were set as follows:

1. The parameters in the STIP detection are the same as those used by Laptev (2005b).
2. To compute the 3SMF feature, the size of the MHI image of video is the same as the image in the video. The size of the MHI image of the XT surface image sequence is *N*_*x*_ × *N*_*t*_. The size of the MHI image of the YT surface image sequence is *N*_*y*_ × *N*_*t*_. Figure 6 shows the MHI of KTH actions.

**Fig. 6:**
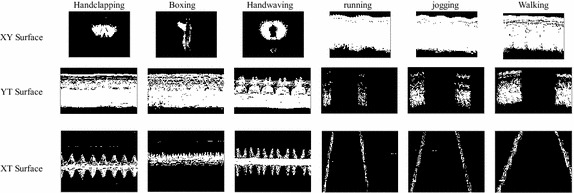
Motion history image of six actions (handclapping, boxing, hand-waving, running, jogging and walking) from three different surfaces. Every MHI belonged to different actions has its own appearance, as shown in *each row*. The same as the above, the MHI of specific action has different appearance in the different surfaces
3. Due to the difference of the length of the video, the sizes of the XTMHI and YTMHI are different, as shown in Fig. 7. The general approach to normalize the XTMHI is image scaling. However, for certain similar actions, image scaling may eliminate the classified information, such as walking, running and jogging. In our study, the length of the video is cut to a fixed length *N*_*c*_ = 200. The same setting is used for YTMHI. The other parameters for detecting 3SMF are set as stated in “Action inference algorithm” section.

**Fig. 7:**
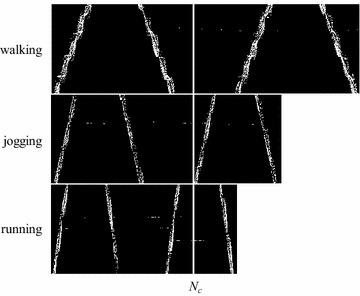
MHI image of the XT surface image sequence (XTMHI)

### Performance evaluation of action recognition

The experimental results of the KTH dataset are shown in Table 1. The comparison results illustrate that the proposed method is effective for human action recognition. The recognition accuracy of the proposed method was the highest among all relevant methods. 3SMF improved the accuracy of action recognition by more than 3 % compared to the approach using STIP and the NBNN algorithm by Zhang et al. (2013).

**Table 1:** Comparison of the proposed method with existing methods for the KTH dataset

Method	Accuracy (%)
3SMF + STIP + NBNN	*96.50*
STIP + NBNN algorithm (uniform distribution) (Zhang et al. 2013)	92.83
Yuan et al. (2011a)	94.00
Yan and Luo (2012)	93.98
Chakraborty et al. (2012)	96.35
Wang et al. (2014)	94.4
Weinland et al. (2010)	92.4

Italic value mean the best results

Comparing with the approach using STIP and the NBNN algorithm in Table 1, we can find it is effective for action recognition by using the 3SMF feature with SVM model to estimate the prior probability instead of uniform distribution. And comparing with other methods on KTH dataset, our approach have the best performance. These results can verify the effective of the proposed method. The confusion matrix of the proposed method is shown in Table 2.

**Table 2:** Confusion matrix of the proposed method on the KTH dataset

	Walking	Running	Jogging	Handwaving	Handclapping	Boxing
Walking	.98	.01	.01	.00	.00	.00
Running	.00	.86	.14	.00	.00	.00
Jogging	.00	.02	.98	.00	.00	.00
Handwaving	.00	.00	.00	.99	.00	.01
Handclapping	.00	.00	.00	.02	.98	.00
Boxing	.00	.00	.00	.00	.00	1.0

In confusion matrix, each column represents the instances in a predict class while each row represents the instances with ground truth. Confusion matrix summarize the classification results of test samples. For example, if there are *N*_*i*_ test samples with action *c*_*i*_, *N*_*i*_ is the number of predict the test samples to action *c*_*j*_ (in KTH dataset, *i* = 6, $N_{i} = \sum\nolimits_{j = 1}^{6} {N_{ij} }$). The value of confusion matrix in first row can been computed as following:

7 $$value_{ij} = \frac{{N_{i} }}{{N_{ij} }}$$

Next, the proposed method was applied to the UCF sport dataset to verify the effectiveness of the proposed method in practical use. Table 3 compares the proposed method to the existing methods. Based on the results, the proposed method has the best accuracy of 94.39 %. Comparing with the best accuracies of the state-of-art methods for the UCF dataset, the improvement of the proposed method is .99 %. Table 4 shows the confusion matrix of the proposed method on the UCF sport dataset.

**Table 3:** Comparison of the proposed method with existing methods for the UCF sports dataset

Methods	Accuracy (%)
3SMF + STIP + NBNN	*94.39*
Wang et al. (2009)	85.60
Yan and Luo (2012)	90.67
Le et al. (2011)	86.50
Shao et al. (2014)	93.4
Zhang et al. (2015)	88.0

Italic value mean the best results

**Table 4:** Confusion matrix of the proposed method on the UCF sport dataset

	Diving	Golf	High-swinging	Kicking	Lifting	Riding	Running	Skating	Swing	Walking
Diving	1.0	.00	.00	.00	.00	.00	.00	.00	.00	.00
Golf	.00	.90	.00	.00	.00	.04	.00	.00	.00	.06
High-swinging	.00	.00	.89	.00	.00	.02	.00	.00	.09	.00
Kicking	.00	.00	.00	1.0	.00	.00	.00	.00	.00	.00
Lifting	.00	.00	.00	.00	1.0	.00	.00	.00	.00	.00
Riding	.00	.00	.00	.00	.00	1.0	.00	.00	.00	.00
Running	.00	.00	.00	.01	.00	.00	.93	.00	.00	.06
Skating	.00	.00	.00	.00	.00	.00	.09	.86	.05	.00
Swing	.00	.00	.00	.00	.00	.00	.05	.00	.95	.00
Walking	.00	.04	.00	.00	.00	.05	.00	.00	.00	.91

Based on these performance evaluation, the accuracy for KTH dataset is close to other methods. For the UCF dataset, the differences are higher. There are two main reasons. Firstly, the proposed 3SMF feature is a holistic feature to representation video. The background of KTH dataset is simple, monotonous and uniformity. And from the confusion matrix in Table 3, we find the classification error occurred mainly in “running” category. This action is very similar with “jogging”. Different with KTH dataset, the special background of UCF dataset is related to respective action category. So the discriminative power of 3SMF for KTH dataset is weaker compared with UCF dataset. Therefore, the improvement of our method of UCF dataset is better than KTH dataset. On the other hand, the accuracy of the existing algorithm for KTH dataset has exceeded 96 %, while only 94 % for UCF dataset. Further improvement has greater challenge in the case of higher accuracy.

## Conclusion

In this paper, we propose a novel multi-surface feature named 3SMF. The prior probability is estimated by an SVM, and the posterior probability is computed by the NBNN algorithm with STIP. We model the relationship score between each video and action as a probability inference to bridge the feature descriptors and action categories. The main contributions of our study is that a new holistic feature (3SMF) is proposed to represent video. 3SMF can reflect the difference of the action in different surfaces. The results of the comparisons with the state-of-the-art action recognition benchmarks demonstrate the effectiveness of the proposed method. However, it also has some limitations in this study. Due to the computation complexity of feature detection and NBNN algorithm, the proposed method is not suitable for real-time recognition. And our method works only in the case that the videos contain one action category. Therefore, in future, we will address the following topics: real-time action recognition and multiple action events recognition in videos.
